# Left ventricular strain and hemodynamic forces from cine CMR for detecting constrictive physiology in pericarditis

**DOI:** 10.1186/s13244-026-02332-2

**Published:** 2026-06-13

**Authors:** Thomas M. Vollbrecht, Annemarie Proff, Sophia Juraschitz, Simon Bienert, Taraneh Aziz-Safaie, Lucia D. Beissel, Leon M. Bischoff, Narine Mesropyan, Dmitrij Kravchenko, Claus C. Pieper, Daniel L. Kuetting, Julian A. Luetkens, Alexander Isaak

**Affiliations:** 1 Department of Diagnostic and Interventional Radiology, University Hospital Bonn, Bonn, Germany; 2 Quantitative Imaging Lab Bonn (QILaB), Bonn, Germany; 3 Department of Neuroradiology, University Hospital Bonn, Bonn, Germany

**Keywords:** pericardium, constrictive pericarditis, magnetic resonance imaging, hemodynamics

## Abstract

**Objectives:**

Constrictive physiology (CP) may complicate pericarditis and requires timely recognition, yet CMR assessment is largely qualitative. This study evaluated the diagnostic value of cine-based strain and hemodynamic forces (HDF) analysis for distinguishing CP from non-constrictive physiology (NCP).

**Materials and methods:**

HDF analysis was retrospectively performed on routine 1.5 T cine images from 2008 to 2024 in patients with CMR-confirmed pericarditis. Patients were classified as CP or NCP non-invasively using guideline-defined clinical and imaging criteria. In addition to left ventricular (LV) function, global longitudinal strain (GLS), global circumferential strain (GCS), and HDF parameters (e.g., apical–basal [A–B] HDF strength) were analyzed. Student’s *t*-test, binary logistic regression, and receiver operating characteristic analysis were performed.

**Results:**

Among 103 patients with pericarditis (mean age 53 ± 18 years), 42 (41%) had CP and 61 (59%) had NCP. LV ejection fraction did not differ between groups, whereas LV end-diastolic volume index was reduced in CP (62.0 ± 16.7 mL/m² vs 75.4 ± 18.2, *p* = 0.003). Strain and HDF parameters were significantly impaired in CP (e.g., A–B HDF strength: 15.3 ± 6.2% vs 21.4 ± 7.3%, *p* < 0.001). LV GLS and A–B HDF strength achieved good diagnostic performance for detecting CP (area under the curve [AUC] 0.76 and 0.78, respectively), further enhanced when combined (AUC 0.82). Both were independent predictors of CP on multivariable analysis (odds ratio [OR] 1.21, *p* = 0.002; and OR 0.83, *p* = 0.007, respectively).

**Conclusions:**

LV strain and HDF analysis using routine CMR cine enabled quantitative detection of CP in pericarditis. A–B HDF strength, particularly in combination with LV GLS, provided the highest diagnostic accuracy.

**Clinical relevance statement:**

Strain and HDF analysis derived from routine cine CMR enable detection of CP in pericarditis, providing novel quantitative parameters that complement conventional qualitative CMR assessment of CP without additional sequences.

**Key Points:**

- CP may complicate pericarditis and can be addressed by targeted therapy, yet CMR diagnosis remains largely qualitative and challenging.
- Novel CMR metrics, including LV GLS and apical–basal HDF, showed good diagnostic performance.
- LV strain and HDF analysis from routine cine CMR cine enabled quantitative detection of CP in pericarditis without additional sequences.

**Graphical Abstract:**

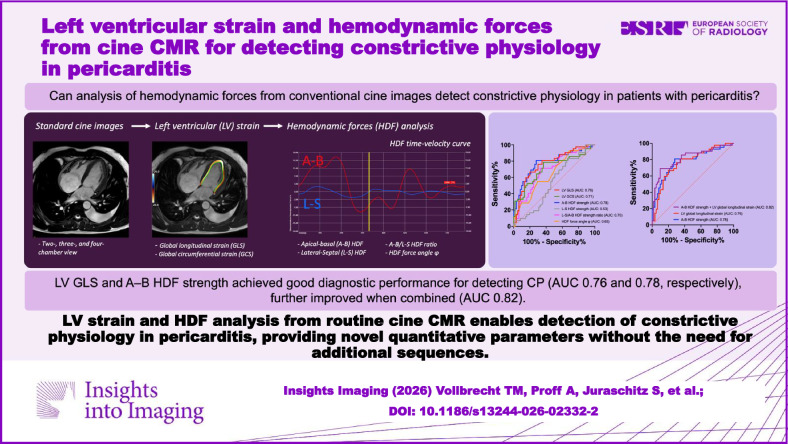

## Introduction

Constrictive physiology (CP) in pericarditis may develop in chronic forms through fibrous thickening and calcification, or in acute forms from transient inelasticity due to inflammation, both leading to restrictive diastolic ventricular filling [1, 2]. The most common causes include idiopathic or viral pericarditis, post-cardiac surgery, post radiation therapy, connective tissue disorders, and post-infectious pericarditis, including tuberculosis [3]. Clinically, patients with pericarditis and CP typically present with signs of right heart failure with preserved ventricular function in the absence of concomitant myocardial disease [4]. However, diagnosis remains challenging, as CP may occur in both acute and chronic phases without heart failure symptoms, pericardial thickening, or calcification [5]. As both transient and chronic forms can resolve with appropriate therapy, ranging from anti-inflammatory treatment to pericardiectomy, developing novel diagnostic parameters remains clinically important.

Cardiovascular magnetic resonance (CMR) is a valuable tool in the diagnostic work-up of pericarditis. Typically following transthoracic echocardiography, multiparametric CMR enables both comprehensive morphological and functional assessment, including pericardial thickness and effusion, ventricular geometry and function, and tissue characterization for inflammatory changes [6]. Furthermore, specific techniques in suspected constriction can be applied, including real-time free-breathing cine images to assess respirophasic septal excursion, free-breathing phase-contrast imaging to quantify respiratory-dependent variations of mitral and tricuspid inflow velocities, and myocardial tagging to detect loss of pericardial-myocardial slippage [7–9]. However, these methods require additional sequences and often yield qualitative assessments that are inherently subjective. Moreover, they depend on adequate patient cooperation and may not be included in standard imaging protocols.

Recently, hemodynamic forces (HDF) emerged as a novel quantitative technique that can be derived from standard cine CMR. HDFs have demonstrated promise in detecting subtle functional changes across a range of cardiac diseases, including heart failure and congenital heart disease [10–16]. Conceptually, HDFs represent the volume integral of intraventricular pressure gradients that drive blood flow throughout the cardiac cycle. While initially quantified invasively via cardiac catheterization, non-invasive methods were later developed using echocardiographic particle image velocimetry and 4D phase-contrast flow imaging, though their widespread use was limited by the need for advanced technology and high-quality imaging [17]. More recently, mathematical models have enabled estimation of HDFs from feature tracking of conventional cine images, without the need for intracardiac flow velocity data [16]. This approach has renewed interest in HDFs, particularly as they appear sensitive to subclinical cardiac dysfunction even before changes in ejection fraction [18]. Since CP limits normal ventricular expansion, we hypothesized that patients with CP would exhibit quantifiable alterations in LV strain and HDFs. Accordingly, the aim of this study was to evaluate the utility of strain and HDF analysis from conventional cine CMR in detecting CP in patients with pericarditis.

## Material and methods

### Study population

This retrospective, observational cohort study included patients with clinically diagnosed and CMR-confirmed pericarditis between March 2008 and December 2024. Clinical diagnosis of pericarditis was established according to the European Society of Cardiology (ESC) criteria [19, 20]. Exclusion criteria were: age ≤ 18 years, incomplete CMR examination, insufficient image quality, CMR diagnosis of cardiac disease other than pericarditis considered more likely (e.g., myocarditis), and general contraindications to contrast-enhanced CMR. Image quality was deemed insufficient for inclusion if any of the following criteria were present: [1] at least one required sequence (e.g., conventional cine or real-time cine) was non-diagnostic due to artifacts (e.g., arrhythmia-related), [2] cine images required for strain or HDF analysis showed incorrect imaging plane orientation, or [3] valve measurements required for HDF analysis could not be performed because of artifacts (e.g., prosthetic valves). Clinical data obtained during hospitalization, including ECG, laboratory results, and echocardiography reports, were reviewed in the hospital information system. No patient had a history of acute coronary syndrome, stroke, or heart failure. In 19 (18%) patients presenting with acute chest pain, immediate catheterization excluded hemodynamically significant coronary artery disease. In accordance with the ESC guideline recommendations, patients were classified as having CP in the presence of impaired diastolic filling on CMR, as defined by diastolic septal bounce and/or respirophasic septal shift [3, 20]. Available information from echocardiography, CT, or cardiac catheterization, as well as clinical symptoms of right heart failure were also recorded to support the classification according to the ESC guidelines [3, 20]. Patients without clinical and imaging features of CP were classified as NCP. This study was approved by the institutional review board with a waiver of informed consent (IRB application number: 095/23-EP).

### Image acquisition

Examinations were performed on a whole-body 1.5 Tesla MRI system (Ingenia 1.5 T, Philips Healthcare). A 16-channel torso coil with digital interface was employed for signal reception. A standardized CMR protocol was applied, comprising retrospectively ECG-gated 2D balanced steady state free precession (bSSFP) cine sequences acquired during breath-hold in short axis (SA) and standard long-axis views (two-, three- and four-chamber) for ventricular anatomy and function, real-time free breathing bSSFP cine images in SA to assess ventricular interdependence, black-blood T2-weighted short tau inversion recovery images to detect inflammatory edema, and T1-weighted inversion-recovery or phase-sensitive inversion recovery sequences for late gadolinium enhancement (LGE) to assess inflammatory enhancement, fibrosis, or scarring. Contrast enhancement was achieved with intravenous gadobutrol or gadoterate meglumine (0.2 mmol/kg body weight; Gadovist, Bayer Healthcare, and Clariscan™, GE Healthcare, respectively). Despite variability in other sequences over time, cine images for strain and HDF analysis were consistently acquired according to contemporary guidelines and remained unchanged in key parameters (e.g., temporal resolution ≤ 45 ms) [21–23].

### Image analysis

All cine images were analyzed offline by T.M.V. (5 years of experience in CMR) under the supervision of A.I. (8 years of experience in CMR) using commercially available software (Medis Suite MR, version 4.0, Medis Medical Imaging Systems B.V.). Readers were blinded to clinical data. LV end-diastolic and end-systolic volumes and ejection fraction were derived from short-axis cine stacks by automated endocardial contouring with manual correction as required. Interventricular septal thickness at diastole (IVSD) was measured manually in the basal inferoseptal segment on cine images in SA. Diastolic septal bounce and respirophasic septal shift were assessed in consensus by the two readers on standard and real-time cine images in SA, respectively. LV strain and HDF analysis were performed using the QStrain module (Medis Suite MR, version 4.0, Medis Medical Imaging Systems B.V). Endocardial and epicardial LV contours were automatically delineated and propagated throughout the cardiac cycle. Tracking accuracy was systematically verified by visual inspection across all cardiac phases. If tracking was deemed inadequate, contours were manually corrected and propagation repeated until satisfactory tracking was achieved throughout the cardiac cycle. Strain parameters included LV global longitudinal (GLS) and global circumferential strain (GCS). HDF analysis was based on endocardial border tracking from strain assessment and manual measurements of the aortic annulus (end-systole) and the mitral annulus (end-diastole) in three-chamber cine images. HDFs were calculated in longitudinal (apical–basal, A–B) and transversal (inferolateral–anteroseptal, L–S) orientations and displayed as time–curve profiles and polar histograms to visualize global LV force distribution. To ensure comparability, HDFs were normalized to LV volume and expressed as a percentage of gravity acceleration. Analyzed parameters included: Root mean squares of A–B and L–S, representing the mean amplitude of HDF strengths during the cardiac cycle; A–B/L–S ratio, representing the relative distribution of HDF direction; and the flow force angle *φ*, representing the orientation of the LV global HDF vector, with 90° indicating a full longitudinal direction.

### Statistical analysis

Prism (version 10.4.1; GraphPad Software) and SPSS (version 29, IBM Corp.) were used for statistical analysis. Categorical variables are presented as absolute numbers and percentages. Continuous variables were tested for normality with the Shapiro–Wilk test and are reported as mean ± standard deviation or as median and interquartile range (IQR), as appropriate. Comparisons between patients with NCP and CP were performed using independent-samples *t*-tests or Fisher’s exact tests, as appropriate. Associations of strain and HDF parameters with CP were assessed by binary logistic regression. Both univariable and multivariable models were calculated; for multivariable analysis, only variables with *p* < 0.05 in univariable logistic regression were entered as candidate predictors, and forward Wald stepwise selection was applied to identify the most relevant predictors. As a sensitivity analysis, we performed an a priori multivariable logistic regression (enter method) including age, sex, LV end-diastolic volume index, and LV ejection fraction to assess whether the main predictors remained associated with CP after adjustment for potential confounders. Diagnostic performance of strain and HDF parameters was evaluated using receiver operating characteristic analysis with calculation of the area under the curve (AUC). Optimal cutoff values were determined using the Youden index. A two-sided *p*-value < 0.05 was considered statistically significant.

## Results

**Fig. 1:**
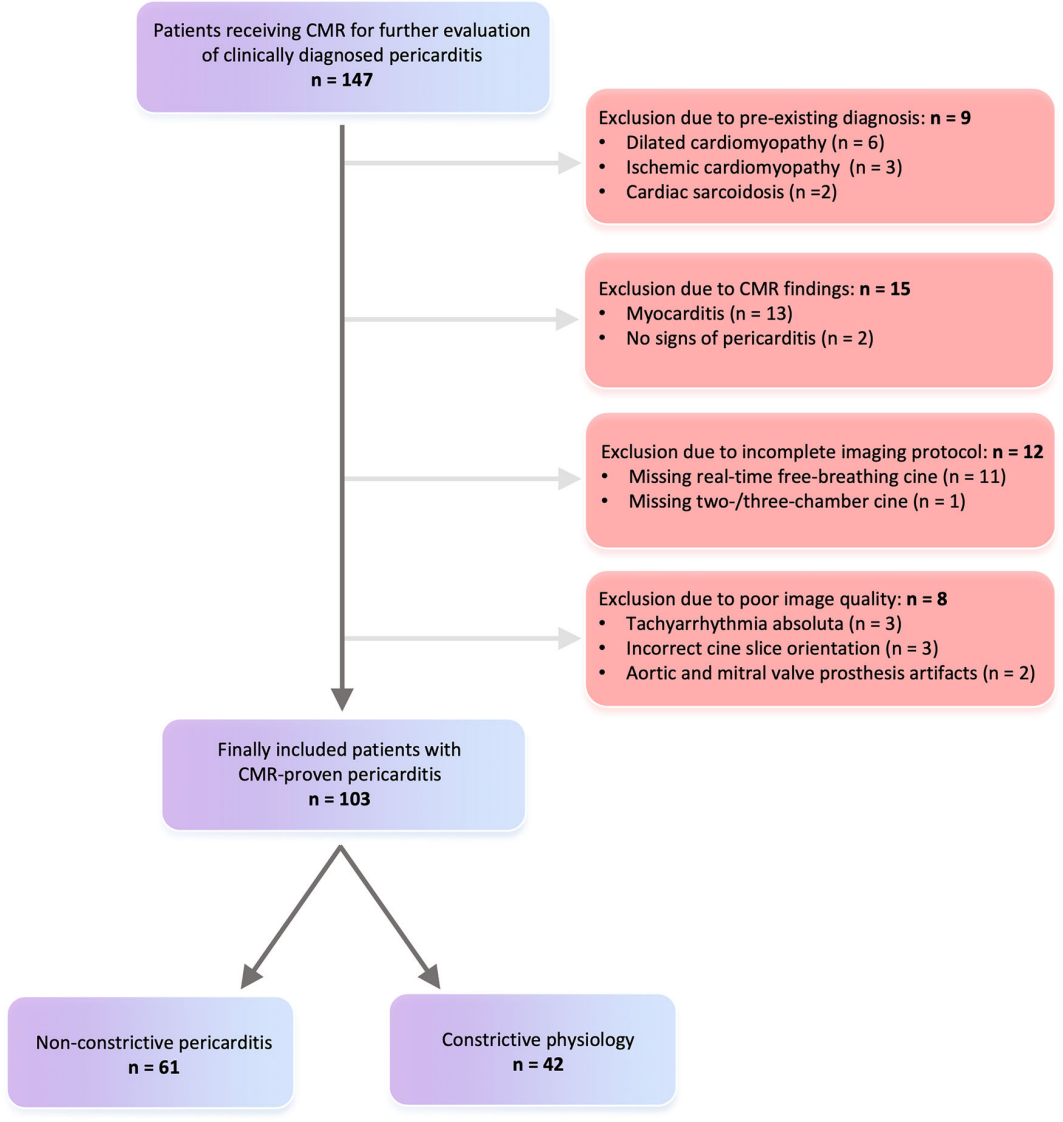
Study flow chart. A total of 103 patients with clinically diagnosed pericarditis were included in the final analysis; 42 (41%) had CP and 61 (59%) had non-constrictive physiology (NCP) based on conventional CMR parameters

A total of 147 consecutive CMR examinations of patients with clinically suspected pericarditis, performed at a tertiary hospital radiology department, were screened for study inclusion. Of these, 44 (30%) were excluded due to pre-existing cardiac conditions potentially confounding HDF analysis (9/44, 20%), CMR findings suggestive of myocarditis rather than pericarditis or absence of pericardial involvement (15/44, 34%), incomplete CMR preventing conventional diagnosis of CP or HDF analysis (12/44, 27%), or poor image quality (8/44, 18%). The final cohort consisted of 103 patients with CMR-proven pericarditis (Fig. 1). Among these, 42 (41%) demonstrated guideline-defined features of CP (i.e., septal bouncing and/or respirophasic septal shift), while 61 of 103 (59%) showed no signs of constriction and were classified as NCP. The most frequent underlying etiologies were autoimmune disorders (27/103, 26%) and infectious disease (19/103, 18%), with 47 of 103 patients (46%) classified as idiopathic. Cardiac enzymes and inflammatory markers were elevated in both groups (e.g., troponin T: 31.7 ± 46.7 ng/l). Detailed clinical characteristics are summarized in Table 1.

**Table 1:** Characteristics of the study population with non-constrictive pericarditis (NCP) and with CP

	All patients *n* = 103	NCP *n* = 61	CP *n* = 42	*p*-value
Age (y)	52.7 ± 18.4	50.4 ± 18.1	56.1 ± 18.4	0.130
Male sex (*n*)	66 (64)	35 (57)	31 (74)	0.099
Body mass index (kg/m^2^)	25.9 ± 4.9	25.2 ± 5.3	26.8 ± 3.9	0.116
Body surface area (mL/m^2^)	1.9 ± 0.3	1.9 ± 0.3	2.0 ± 0.2	0.336
Etiology				
Infectious (*n*)	19 (18)	9 (15)	10 (24)	0.304
Autoimmune (*n*)	27 (26)	17 (28)	10 (24)	0.820
Neoplastic (*n*)	2 (2)	2 (3)	0 (0)	0.512
Traumatic and Iatrogenic (*n*)	8 (8)	5 (8)	3 (7)	> 0.999
Idiopathic (*n*)	47 (46)	28 (46)	19 (45)	> 0.999
Symptom onset to CMR (*d*)	20 (7–41)	15 (7–48)	27 (9–37)	0.733
Acute (< 30 days)	44 (43)	31 (51)	13 (31)	0.068
Subacute (30–90 days)	28 (27)	15 (25)	13 (31)	0.506
Chronic (> 90 days)	31 (30)	15 (25)	16 (38)	0.190
Clinical symptoms				
Acute chest pain (*n*)	44 (48)	28 (53)	16 (42)	0.396
Dyspnea (*n*)	45 (44)	16 (26)	29 (69)	**< 0.001**
Signs of right heart failure (*n*)	42 (41)	10 (16)	32 (76)	**< 0.001**
Palpitations/arrhythmia (*n*)	17 (17)	9 (15)	8 (19)	0.598
Pericardial friction rubs (*n*)	17 (17)	13 (21)	4 (10)	0.176
ECG alterations (*n*)	37 (45)	21 (41)	16 (50)	0.499
Laboratory testing				
Trop T (ng/L)	31.7 ± 46.7	37.5 ± 55.9	21.9 ± 20.1	0.243
NT pro-BNP (pg/mL)	1722.5 ± 4023.0	1299.6 ± 1960.4	2303.9 ± 5707.1	0.461
C-reactive protein (mg/L)	84.5 ± 77.4	77.9 ± 69.2	93.5 ± 86.6	0.369
Leucocytes (10^3^/μmL)	10.9 ± 4.3	10.9 ± 4.0	10.9 ± 4.7	0.988
Pericardial effusion (*n*)	75 (73)	47 (77)	28 (67)	0.267
Non-CMR imaging consistent with CP*				
Echocardiography (*n*)	-	-	32 (76)	-
CT (*n*)	-	-	8 (19)	-
Cardiac catheterization (*n*)	-	-	6 (14)	-

Data are numbers (%) of cases, means ± standard deviation, or median and IQRs. Bold indicates statistical significance (*p* < 0.05)

*Non-CMR imaging findings consistent with CP followed the current ESC guidelines (3)

### CMR characteristics

**Fig. 2:**
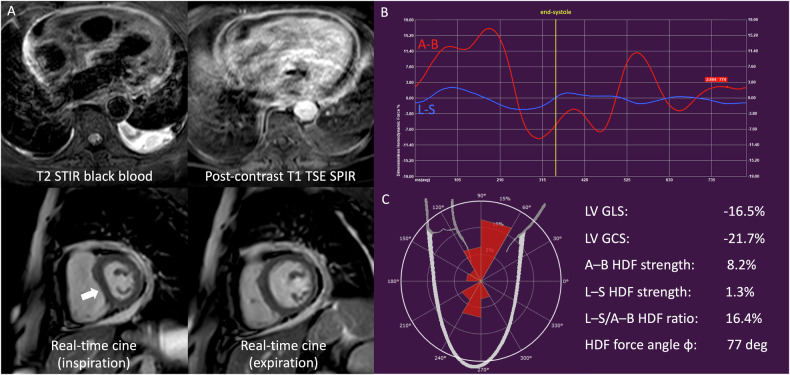
Clinical example of pericarditis with CP in a 70-year-old woman presenting with right heart failure. **A** T2 short-tau inversion recovery (STIR) black-blood imaging shows circumferential edematous pericardial thickening, and post-contrast T1 post-contrast turbo spin-echo spectral presaturation with inversion recovery (SPIR) reveals circumferential pericardial enhancement. Real-time short-axis cine images with dynamic respiration demonstrate leftward septal excursion during deep inspiration (white arrow), indicating ventricular interdependence, which normalized during expiration. **B** HDF time profiles from apical-basal and lateral-septal components (A, B HDF strength, red line, and L–S HDF strength, blue line, respectively) demonstrate low mean amplitudes throughout the cardiac cycle, with particularly reduced longitudinal A, B HDF strength. **C** Corresponding polar histogram indicates slight deviation from physiological longitudinal alignment towards pronounced transversal orientation, reflected by an elevated L–S/A–B HDF ratio (16.4%) and HDF force angle (φ = 77°). Also, left ventricular (LV) GLS (−16.5%) and LV GCS (−21.7%) were significantly impaired in this patient

**Fig. 3:**
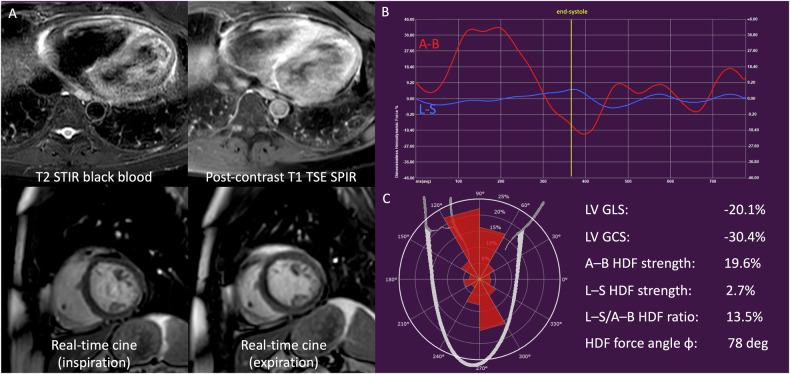
Clinical example of NCP in a 45-year-old male patient presenting with respiratory-dependent acute chest pain. **A** T2 STIR black-blood imaging demonstrates circumferential pericardial edema, while post-contrast T1 post-contrast turbo spin-echo SPIR reveals circumferential pericardial enhancement. Real-time short-axis cine images with dynamic respiration show normal septal movement during inspiration and expiration without signs of constriction. **B** HDF time profiles from apical-basal and lateral-septal components (A–B HDF strength, red line, and L–S HDF strength, blue line, respectively) demonstrate preserved mean amplitudes throughout the cardiac cycle (A–B HDF strength 19.6%, L–S HDF strength 2.7%). C) Corresponding polar histogram illustrates inconspicuous orientation from the apex toward the LV outflow tract, (L–S/A–B HDF ratio 13.5%, HDF force angle φ = 78 degrees). Compared to patients with CP, LV GLS and LV GCS were preserved

CMR was performed after a median of 20 days (IQR, 7–41 days) after symptom onset, with no significant difference between patients with CP and NCP (27 [9–37] days vs 15 [7–48] days, *p* = 0.733). Heart rates during scanning were comparable between groups (75 ± 12 bpm vs 77 ± 19 bpm, *p* = 0.678). Patients with CP had significantly lower LV indexed end-diastolic volumes compared to NCP patients (62.0 ± 16.7 vs 75.4 ± 18.2, *p* = 0.003), while LV ejection fraction did not differ (59.5 ± 6.9% vs 58.6 ± 7.9%, *p* = 0.173). Prevalence and extent of pericardial effusion were comparable in patients with CP and NCP (28/42 [67%] vs 47/61 [77%], *p* = 0.267, and 13.7 ± 9.3 mm vs 14.2 ± 9.8 mm, *p* = 0.115, respectively). Also, prevalence of pericardial edema did not differ between both groups (33/42 [79%] vs 51/61 [84%], *p* = 0.608). LGE of the pericardium was observed in 41 of 42 (98%) patients with CP and in all patients with NCP (*p* = 0.408); in CP, LGE involvement of the global pericardium was significantly more frequent (37/42 [90%] vs 45/61 [74%], *p* = 0.045), whereas segmental involvement was less frequent (4/42 [10%] vs 16/61 [26%]). Additional non-ischemic LGE of the myocardium indicating pericarditis with myocardial involvement was comparable between groups (4/42 [10%] vs 8/61 [13%], *p* = 0.757). LV GLS and GCS were significantly impaired in patients with CP compared to patients with NCP (−18.1 ± 4.6% vs −22.2 ± 3.9%, *p* < 0.001, and −27.2 ± 6.8% vs −31.9 ± 5.4%, *p* < 0.001, respectively). HDF analysis showed reduced A–B HDF strength (15.3 ± 6.2% vs 21.4 ± 7.3%, *p* < 0.001), increased L–S/A–B ratio (29.6 ± 10.4% vs 23.1 ± 9.2%, *p* = 0.001), and a smaller HDF force angle in patients with CP compared to patients with NCP (69.7 ± 5.1° vs 72.4 ± 4.9°, *p* = 0.008). L–S HDF strength did not differ between the two groups (Table 2). Representative clinical examples of CP and NCP are shown in Figs. 2 and 3, respectively.

**Table 2:** CMR characteristics of the study population with non-constrictive pericarditis (NCP) and with CP

	NCP *n* = 61	CP *n* = 42	*p*-value
Morphology and function			
Heart rate (bpm)	77 ± 19	75 ± 12	0.678
LV ejection fraction (%)	58.6 ± 7.9	59.5 ± 6.9	0.173
LV end-diastolic volume (mL)	147.9 ± 43.6	124.5 ± 38.7	**0.035**
LV end-diastolic volume index (mL/m^2^)	75.4 ± 18.2	62.0 ± 16.7	**0.003**
Interventricular septal thickness at diastole (mm)	9.3 ± 2.2	9.0 ± 1.5	0.343
Pericardial thickness (*n*)	4.6 ± 1.4	5.7 ± 2.1	**0.003**
Pericardial effusion thickness (*n*)	14.2 ± 9.8	13.7 ± 9.3	0.115
Aortic valve (mm)	22.8 ± 2.9	23.5 ± 2.6	0.225
Mitral valve (mm)	34.1 ± 3.7	35.3 ± 5.1	0.175
Conventional signs of CP			
Diastolic septal bounce (*n*)	-	34 (81)	-
Respirophasic septal excursion (*n*)	-	38 (90)	-
Tissue characterization			
Pericardial edema (*n*)	51 (84)	33 (79)	0.608
Segmental (*n*)	10 (20)	4 (12)	0.550
Global (*n*)	41 (80)	29 (88)	0.550
Pericardial LGE (*n*)	61 (100)	41 (98)	0.408
Segmental (*n*)	16 (26)	4 (10)	**0.045**
Global (*n*)	45 (74)	37 (90)	**0.045**
Myocarditis-pattern LGE (*n*)	8 (13)	4 (10)	0.757
LV myocardial strain			
GLS (%)	−22.2 ± 3.9	−18.11 ± 4.6	**< 0.001**
GCS (%)	−31.9 ± 5.4	−27.2 ± 6.8	**< 0.001**
HDF			
A-B (%)	21.4 ± 7.3	15.3 ± 6.2	**< 0.001**
L-S (%)	4.9 ± 2.7	4.2 ± 1.5	0.145
L-S/A-B ratio	23.1 ± 9.2	29.6 ± 10.4	**0.001**
HDF force angle φ (deg)	72.4 ± 4.9	69.7 ± 5.1	**0.008**

Data are numbers (%) of cases or means ± standard deviation. Bold indicates statistical significance (*p* < 0.05)

*LGE* late gadolinium enhancement, *LV* left ventricular, *RMS* root mean square

### Association of LV strain and HDF with CP

Univariable binary logistic regression analysis demonstrated that the presence of CP was associated with LV GLS and GCS, as well as HDF parameters including A–B HDF strength, L–S/A–B ratio, and HDF force angle (e.g., A–B: Odds ratio [OR], 0.84; 95% confidence interval [95% CI]: 0.77–0.92; *p* < 0.001) (Table 3). No significant association was observed between CP and L–S HDF strength (OR, 0.86; 95% CI: 0.71–1.06; *p* = 0.153). All strain and HDF parameters were entered in a multivariable binary logistic regression model using forward Wald selection. In the final model, LV GLS and A–B HDF strength remained independent predictors of CP (OR, 1.21; 95% CI: 1.07–1.38; *p* = 0.003, and OR, 0.89; 95% CI: 0.82–0.97; *p* = 0.005, respectively). In an a priori confounder-adjusted sensitivity analysis including age, sex, LV end-diastolic volume index, and LV ejection fraction, LV GLS (OR, 1.27; 95% CI: 1.07–1.51; *p* = 0.006) and A–B HDF strength (OR, 0.89; 95% CI: 0.81–0.98; *p* = 0.014) remained independently associated with CP.

**Table 3:** Influence of HDF and LV strain parameters for the prediction of the presence of CP in pericarditis

	Univariable analysis		Multivariable analysis	
	OR (95% CI)	*p*-value	OR (95% CI)	*p*-value
LV myocardial strain				
GLS	1.26 (1.12–1.41)	**< 0.001**	1.21 (1.07–1.38)	**0.003**
GCS	1.15 (1.06–1.25)	**< 0.001**	…	…
HDF				
A–B	0.84 (0.77–0.92)	**< 0.001**	0.89 (0.82–0.97)	**0.005**
L–S	0.86 (0.71–1.06)	0.153	…	…
L–S /A–B ratio	1.07 (1.02–1.12)	**0.003**	…	…
HDF force angle φ	0.90 (0.83–0.98)	**0.011**	…	…

Data in parentheses are 95% CIs. *p*-values were calculated using binary logistic regression. Variables for the multivariable model were selected by forward stepwise selection (Wald). Bold indicates statistical significance (*p* < 0.05)

### Diagnostic performance of LV strain and HDF parameters

**Fig. 4:**
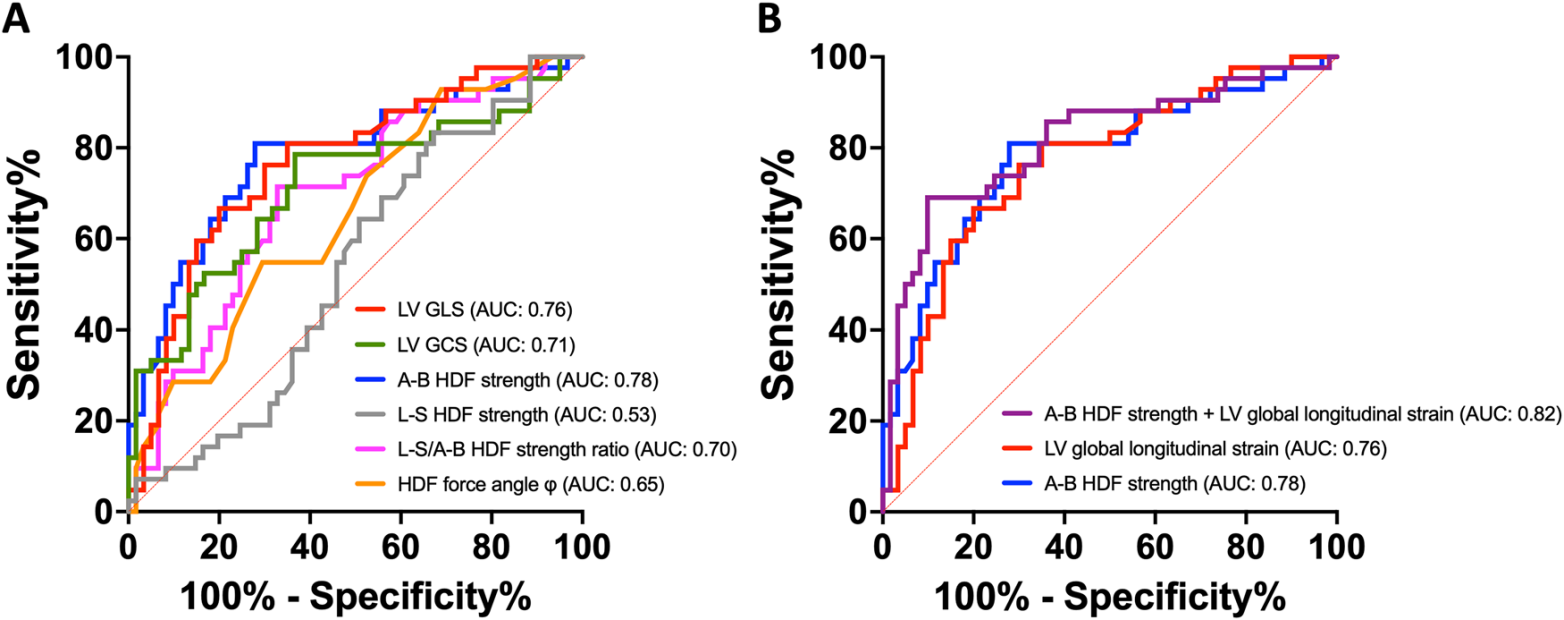
**A** Graph shows receiver operating characteristic curves for different strain parameters: LV GLS: area under the receiver operating characteristic curve [AUC], 0.70; LV GCS: AUC, 0.71; apical-basal HDF (A–B HDF) strength: AUC, 0.78; lateral-septal HDF (L–S HDF) strength: 0.53; L–S/A–B HDF strength ratio: AUC, 0.70; and HDF force angle φ: AUC, 0.65. **B** Graph shows receiver operating characteristic curves for LV GLS (AUC, 0.76), for A–B HDF strength (AUC, 0.78), and for their respective combination (AUC, 0.82)

Among LV strain and HDF parameters, highest diagnostic performance was observed for LV GLS, using an optimal cutoff of >−19.5% (AUC, 0.76; 95% CI: 0.67–0.86; sensitivity, 67%; 95% CI: 52–79%; specificity, 80%; 95% CI: 68–88%), and A–B HDF strength, using an optimal cutoff of < 18.1% (AUC, 0.78; 95% CI: 0.69–0.88; sensitivity, 81%; 95% CI: 67–90%; specificity, 80%; 95% CI: 60–82%). Diagnostic performance was further improved by combining LV GLS and A–B HDF strength based on predicted probabilities from multivariable logistic regression (AUC, 0.82; 95% CI: 0.73–0.90; sensitivity, 69%; 95% CI: 54–81%; specificity, 90%; 95% CI: 80–95%). AUC values, cutoff values, sensitivities, and specificities for all evaluated parameters are summarized in Table 4, with corresponding receiver operating characteristic curves shown in Fig. 4.

**Table 4:** Diagnostic performance of HDF and LV strain parameters for the diagnosis of CP in pericarditis

	AUC	Cutoff	Sensitivity (%)	Specificity (%)
LV myocardial strain				
GLS	0.76 (0.67–0.86)	> −19.5	67 (52–79)	80 (68–88)
GCS	0.71 (0.61–0.82)	> −31.0	79 (64–88)	63 (51–74)
HDF				
A–B	0.78 (0.69–0.88)	< 18.1	81 (67–90)	72 (60–82)
L–S	0.53 (0.42–0.65)	< 5.7	83 (69–92)	33 (32–45)
L–S/A–B ratio	0.70 (0.59–0.80)	> 24.6	71 (56–83)	67 (55–78)
HDF force angle φ	0.65 (0.55–0.76)	< 71	55 (40–69)	70 (58–80)

Values in parentheses are 95% CIs, and values in square brackets are numerators and denominators for percentages

*AUC* area under the receiver operating characteristic curve

## Discussion

This study evaluated LV strain and HDF parameters derived from conventional CMR cine imaging for the detection of CP in pericarditis. Differences between patients with CP and NCP were found for LV GLS and GCS, A–B HDF strength, L–S/A–B ratio, and HDF force angle. Among individual parameters, LV GLS and A–B HDF strength emerged as independent predictors of CP and demonstrated the highest diagnostic performance, which was further improved in combination.

While LV myocardial strain imaging by CMR feature tracking is already an established technique for the assessment of myocardial deformation across a wide variety of cardiac diseases [24], HDF analysis, building upon feature tracking and mathematical modeling, represents a novel quantitative tool that characterizes intraventricular pressure gradients generated by the cyclic interaction between myocardial interaction and blood flow [25]. Conceptually, it can therefore be regarded as the fluid-dynamic correlate of deformation imaging [18]. In alignment with previous studies conducted in patients with heart failure, the A–B HDF strength, which reflects the mean amplitude of the longitudinal HDF throughout the cardiac cycle, demonstrated the best discriminative performance among HDF parameters in the present study [10, 26]. This can be attributed to the LV geometry, as the dominant direction of myocardial fiber shortening and blood propulsion are both nearly consistent with the longitudinal axis [27]. In the setting of CP, the underlying pathophysiological alterations also predominantly affect the longitudinal axis of the LV. Increased pericardial stiffness restricts diastolic filling, which can secondarily increase the effective systolic burden (e.g., via compensatory vasoconstriction in response to reduced LV volume) and alter the normal pattern of myocardial deformation, potentially leading to impaired systolic performance despite preserved global ejection fraction [17]. Accordingly, both LV GLS and A–B HDF strength were markedly reduced in patients with CP compared to those without constriction, supporting their potential role as markers of altered longitudinal mechanics. By contrast, L–S HDF strength was not significantly altered by CP. This relative preservation of transverse dynamics in the presence of impaired longitudinal function resulted in maladaptive redistribution of force vectors, as reflected by an increased L–S/A–B ratio and reduced HDF force angle. Taken together, these alterations suggest inefficient coupling of longitudinal and transverse HDF components, representing a loss of intraventricular synchrony and energy efficiency, and providing further mechanistic explanation for adverse ventricular physiology in constriction.

While HDF analysis represents a novel application in CMR of pericarditis, previous studies have already focused on LV strain in CP, mainly for differentiating CP from restrictive cardiomyopathy [28–31]. Consistent with our findings, disrupted LV strain performance was found in CP, particularly in the longitudinal direction [28, 30]. Our results demonstrate that both strain and HDF parameters can also discriminate CP within a cohort of patients with clinically diagnosed pericarditis. Among these, LV GLS and A–B HDF strength were independent predictors with comparable overall diagnostic performance yet distinct profiles; while A–B HDF strength provided higher sensitivity (81%) but lower specificity (72%), global GLS showed lower sensitivity (67%) but higher specificity (80%). Thus, while derived from the same feature tracking framework, strain and HDF may reflect different aspects of ventricular function and therefore should not be considered interchangeable. While strain reflects mechanical deformation of the myocardium, HDF captures the pressure-related driving forces of cardiac motion. Combined assessment may therefore provide a more comprehensive understanding in pericarditis by bridging myocardial mechanics to intraventricular hemodynamics, linking structural constraints to functional consequences.

Diagnosis of CP has traditionally been challenging, as clinical symptoms often overlap with those of other cardiac diseases. In recent years, advances in multimodality imaging have improved diagnostic accuracy [6, 32].

CMR has evolved as a key pillar in diagnosing CP, providing a comprehensive assessment of both structural and functional aspects. In addition to dynamic visualization of impaired diastolic filling through septal bounce and respirophasic septal shift, supportive features include morphologic alterations such as pericardial thickening [33].

Importantly, assessment of conventional parameters associated with constriction often requires additional sequences, such as real-time cine, free-breathing phase-contrast imaging, or myocardial tagging. These can be challenging to obtain in patients with limited compliance or reduced respiratory capacity and may not be included in routine CMR protocols, thereby restricting their applicability and limiting retrospective evaluation. In contrast, strain and HDF parameters can be derived from standard cine images, avoiding prolonged scan times and enabling retrospective analysis even when specialized sequences are unavailable. Post-processing is largely automated using commercially available software and requires only minor manual input, which may facilitate clinical implementation [34].

In addition, most conventional imaging markers of constriction, such as septal bounce or respirophasic septal shift, are primarily qualitative and yield binary assessments (present/absent) that are clinically valuable for decision-making. However, they do not quantify functional severity and may therefore be susceptible to over- or underestimation of physiological impact. Quantitative parameters such as strain and HDF metrics may help characterize the degree of functional impairment, for example, by reflecting deviation from reference or threshold values, and could also facilitate longitudinal comparison during follow-up.

Taken together, strain and HDF parameters may provide additional quantitative information alongside conventional CMR markers and thus could offer complementary insight into the functional consequences of constriction rather than replacing established diagnostic criteria. Based on these exploratory findings, future studies are warranted to determine whether these parameters improve diagnostic confidence in borderline cases, enable grading of disease severity, or support monitoring of treatment response.

### Limitations

This study is limited by its retrospective and single-center design, limiting the generalizability of the results. The presence of CP was not confirmed by invasive hemodynamic assessment or pericardial biopsy in most cases, but was determined based on CMR and other imaging modalities in accordance with current guideline recommendations. In clinical practice, both cardiac catheterization and pericardial biopsy are rarely performed in pericarditis and are reserved for selected cases [3]. CP was relatively frequent in this cohort, which may reflect selection bias, as exclusion of examinations without real-time cine may have preferentially removed clinically milder cases without CP. Furthermore, the diagnostic performance of novel CMR parameters was evaluated using established CMR markers from the same scans as reference, which may have introduced bias due to modality-internal validation. However, the primary aim was to investigate strain and HDF as complementary tools to conventional CMR parameters, not as full replacements requiring head-to-head comparison. Finally, due to the retrospective design, standardized follow-up data were not available, and the prognostic value of the investigated parameters could therefore not be assessed.

### Conclusion

Strain and HDF analysis derived from standard cine CMR enables detection of CP in pericarditis, with A–B HDF strength demonstrating the highest diagnostic performance, particularly when combined with LV GLS. As no additional sequences are needed, implementation of HDF analysis appears feasible and may add quantitative information to conventional qualitative CMR assessment of pericarditis.

## Data Availability

The data analyzed during this study are available from the corresponding author upon reasonable request.

## Abbreviations

95% CI: 95% confidence interval
CMR: Cardiovascular magnetic resonance
CP: Constrictive physiology
ESC: European Society of Cardiology
GCS: Global circumferential strain
GLS: Global longitudinal strain
IQR: Interquartile range
LV: Left ventricular
NCP: Non-constrictive physiology
OR: Odds ratio
SA: Short axis
